# Influence of Molecular Structure on O_2_-Binding Properties and Blood Circulation of Hemoglobin‒Albumin Clusters

**DOI:** 10.1371/journal.pone.0149526

**Published:** 2016-02-19

**Authors:** Kana Yamada, Kyoko Yokomaku, Risa Haruki, Kazuaki Taguchi, Saori Nagao, Toru Maruyama, Masaki Otagiri, Teruyuki Komatsu

**Affiliations:** 1 Department of Applied Chemistry, Faculty of Science and Engineering, Chuo University, 1-13-27, Kasuga, Bunkyo-ku, Tokyo, 112–8551, Japan; 2 Faculty of Pharmaceutical Sciences, Sojo University, 4-22-1, Ikeda, Nishi-ku, Kumamoto, 860–0082, Japan; 3 Department of Biopharmaceutics, Graduate School of Pharmaceutical Sciences, Kumamoto University, 5–1 Oe-Honmachi, Chuo-ku, Kumamoto, 862–0973, Japan; Russian Academy of Sciences, Institute for Biological Instrumentation, RUSSIAN FEDERATION

## Abstract

A hemoglobin wrapped covalently by three human serum albumins, a Hb-HSA_*3*_ cluster, is an artificial O_2_-carrier with the potential to function as a red blood cell substitute. This paper describes the synthesis and O_2_-binding properties of new hemoglobin‒albumin clusters (i) bearing four HSA units at the periphery (Hb-HSA_*4*_, large-size variant) and (ii) containing an intramolecularly crosslinked Hb in the center (XLHb-HSA_*3*_, high O_2_-affinity variant). Dynamic light scattering measurements revealed that the Hb-HSA_*4*_ diameter is greater than that of either Hb-HSA_*3*_ or XLHb-HSA_*3*_. The XLHb-HSA_*3*_ showed moderately high O_2_-affinity compared to the others because of the chemical linkage between the Cys-93(β) residues in Hb. Furthermore, the blood circulation behavior of ^125^I-labeled clusters was investigated by assay of blood retention and tissue distribution after intravenous administration into anesthetized rats. The XLHb-HSA_*3*_ was metabolized faster than Hb-HSA_*3*_ and Hb-HSA_*4*_. Results suggest that the molecular structure of the protein cluster is a factor that can influence *in vivo* circulation behavior.

## Introduction

In Japan, 85% of blood products are used for patients who are over 50 years old [1]. Japan’s continuously declining birthrate and aging society make it difficult to retain a stable blood transfusion system. The Japanese Red Cross Society forecasts a severe blood shortage equivalent to 890,000 people per year in 2027 [2]. Consequently, a blood alternative, especially a red blood cell (RBC) substitute, is necessary as a medical measure to complement blood transfusion treatment. In the last few decades, hemoglobin (Hb)-based O_2_-carriers (HBOCs) of various kinds, such as glutaraldehyde-polymerized Hb [3–6] and poly(ethyleneglycol)-conjugated Hb (PEG-Hb) [7–11], have been developed [12,13]. However, the practical use of a safe and effective formulation has not been realized [3,5,6,12,14,15]. A main side-effect is vasoconstriction, which elicits an acute increase in blood pressure. Many investigators have hypothesized that the vasopressor response is attributable to rapid quenching of nitric oxide (endothelial-derived relaxing factor) by a small fraction of free Hb leaked into the extravascular space [16–18].

**Fig 1:**
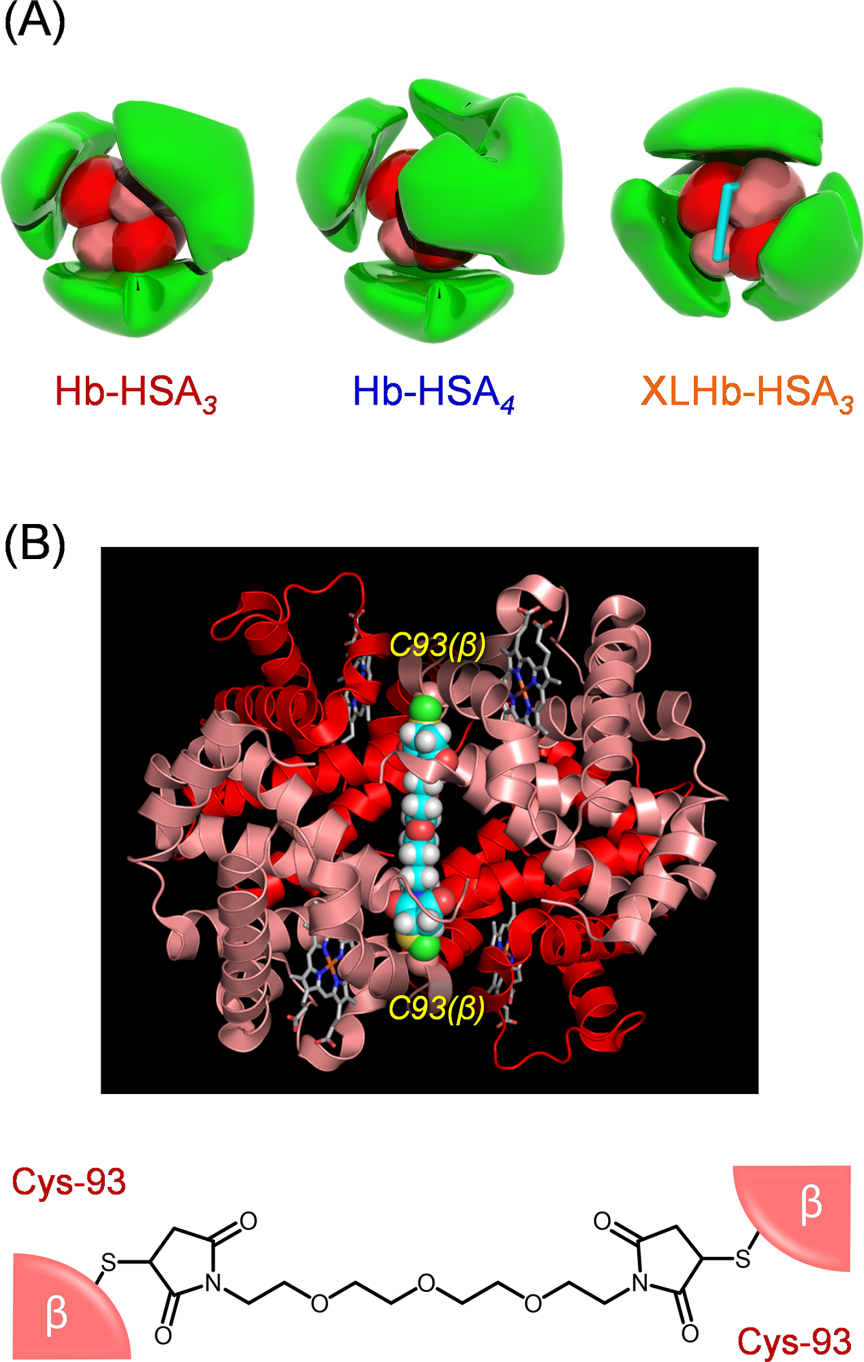
Schematic illustrations of hemoglobin-albumin clusters. (A) Molecular structures of Hb-HSA_*3*_, Hb-HSA_*4*_, and XLHb-HSA_*3*_. (B) Structural model of intramolecularly crosslinked Hb, in which sulfhydryl groups of Cys-93(β) residues (distance: 21.6 Å) are connected by BMTEG (molecular length: 18.3 Å, shown in a space-filling representation) [25,26].

Human serum albumin (HSA), the major plasma protein in the bloodstream (approximately 0.6 mM), fills two crucial roles of maintaining colloid osmotic pressure and of transporting hydrophobic metabolites and drugs [19]. We demonstrated previously that a covalent core–shell structured protein cluster comprising Hb in the center and HSA at the periphery functions as a unique HBOC [20–24]. The average binding number of HSA on one Hb is 3.0 ± 0.2. We designate this cluster as Hb-HSA_*3*_ (Fig 1A). HSA contains only one free sulfhydryl group at Cys-34. Consequently, a heterobifunctional crosslinker, succinimidyl-4-(*N*-maleimidomethyl)cyclohexane-1-carboxylate (SMCC) was used for coupling of the Cys-34 of HSA with surface amino groups of Lys residues of Hb [20,21]. The resultant cluster has satisfactorily negative surface net charge (*p*I: 5.1) because of the surrounding HSA shells. As expected, intravenous transfusion of Hb-HSA_*3*_ into anesthetized rats (i) did not invoke unfavorable systemic hypertension but (ii) showed reasonably long blood circulation [24]. Such superior properties of Hb-HSA_*3*_ are a consequence of the electrostatic repulsion between the cluster surface and glomerular basement membrane around the endothelial cells. If one were able to engineer the binding number of exterior HSAs and the chemical structure of interior Hb, then it might engender unique variants with different molecular structure, molecular size, O_2_-affinity, and blood retention. In this study, we designed and synthesized new hemoglobin‒albumin clusters bearing four HSA units at the periphery (Hb-HSA_*4*_, large-size variant) and including an intramolecularly crosslinked Hb (XLHb) in the center (XLHb-HSA_*3*_, high O_2_-affinity variant) (Fig 1A). The influences of molecular structure on O_2_-binding properties and blood circulation of the protein cluster have been investigated.

## Materials and Methods

### Materials and Apparatus

Human serum albumin (HSA) was purchased from the Japan Blood Products Organization. Hemoglobin (Hb) was purified from bovine red blood cells purchased from Tokyo Shibaura Zouki Co. Ltd. Succinimidyl-4-(*N*-maleimidomethyl)cyclohexane-1-carboxylate (SMCC) was purchased from Wako Pure Chemical Industries Ltd. 1,11-Bismaleimido-triethyleneglycol (BMTEG) was purchased from Thermo Fischer Scientific K.K. The water was deionized (18.2 MΩcm) using water purification systems (Elix UV and Milli Q Reference; Millipore Corp.). Native-PAGE and SDS-PAGE were conducted using an electrophoresis power supply (EPS 301; GE Healthcare) with precast 5‒20% polyacrylamide gel (Hi-QRAS Gel N 5‒20%: Kanto Chemical Co. Inc.). Isoelectric focusing (IEF) was performed using an electrophoresis power supply (EPS 601; GE Healthcare) with a pH 3–10 IEF protein gel (Novex; Thermo Fischer Scientific K.K.). The voltage was raised gradually to 500 V for 2 h.

### Synthesis of Hb-HSA_*4*_ Cluster

DMSO solution of SMCC (40 mM, 2.0 mL) was added dropwise to the carbonyl Hb solution (0.1 mM, 20 mL in phosphate buffered saline solution, PBS) in a round-bottom flask (50-mL volume). Then the mixture was stirred under dark conditions in CO atmosphere for 2 h at 4°C. The resultant solution was loaded directly onto a gel filtration chromatograph (GFC) with a Sephadex G25 (superfine) column to remove unreacted SMCC. The eluent was concentrated to 20 mL ([Hb] = 0.1 mM) using a centrifugal concentrator (Vivaspin 20 ultrafilter, 10 kDa MWCO; Sartorius AG). The obtained maleimide-activated-Hb (0.1 mM, 20 mL) was added slowly into the HSA solution (2.0 mM, 20 mL in PBS). Then the mixture was stirred for 14 h under dark conditions at 4°C. Part of the reactant was applied for size-exclusion chromatography (SEC) on an HPLC system (LaChrom Elite; Hitachi High-Technologies Corp.) with a column (Shodex Protein KW-803; Showa Denko K.K.) using phosphate buffered (PB) solution (50 mM, pH 7.4) as the mobile phase. The elution curve showed a new broad peak at the high molecular weight region. Then the resultant solution was subjected to GFC (Superdex 200 pg; GE Healthcare UK Ltd.) using PBS as the running buffer. The eluent was monitored at 280 nm, and the cluster fraction was collected. The total protein concentration and Hb concentration were measured, respectively, using a protein assay kit (Pierce 660 nm; Thermo Fisher Scientific K.K.) and Hb assay kit (Nescoat Hemokit-N; Alfresa Pharma Corp.). The cysteinyl thiol assay of Hb was conducted by reaction with 4,4’-dithiopyridine, which binds sulfhydryl group of the protein to give 4-thiopyridinone (*λ*_max_: 324 nm) [27]. The collected yield was ca. 80% based on Hb. The average HSA/Hb ratio of the product was 4.0 ± 0.2, which is indicated as Hb-HSA_*4*_ with italicized subscript 4. The metHb ratio of Hb-HSA_*4*_ was less than 1%.

### Synthesis of XLHb-HSA_*3*_ Cluster

DMSO solution of 1,11-bismaleimido-triethyleneglycol (BMTEG) (10 mM, 1.0 mL) was added dropwise to the carbonyl Hb solution (0.1 mM, 10 mL in PBS) in a round-bottom flask (30-mL volume). After stirring for 1 h in CO atmosphere under dark conditions at 4°C, the reactant was loaded onto GFC with a Sephadex G25 (superfine) column to remove unreacted BMTEG. The eluent of ββ-crosslinked Hb (XLHb) was concentrated to 10 mL ([XLHb] = 0.1 mM) using a centrifugal concentrator (Vivaspin 20 ultrafilter, 10 kDa MWCO). SDS-PAGE exhibited two bands at 16 kDa and 32 kDa, which respectively correspond to the α-subunit monomer and crosslinked ββ-subunit dimer. The cysteinyl thiol assay of XLHb using 4,4’-dithiopyridine showed that both thiol groups of Cys-93(β) of Hb were bridged by BMTEG.

XLHb-HSA_*3*_ was prepared according to the same procedure used for Hb-HSA_*3*_ using XLHb [21]. The collected yield was ca. 80% based on Hb. The average HSA/XLHb ratio of the cluster mixture was 2.9 ± 0.2, which is denoted as XLHb-HSA_*3*_ with italicized subscript 3.

### DLS Measurements

The molecular sizes of the protein clusters were ascertained using dynamic light scattering (DLS) measurements (Zetasizer Nano ZS; Malvern Instruments, Ltd.) at 25°C. A He-Ne laser (633 nm) was used and the scattering angle was 173°. Aqueous protein solutions with a concentration of 5 μM were prepared in Milli-Q water. The hydrodynamic diameters were estimated by number from the particle size distribution.

### O_2_-Binding Property

The visible absorption spectra were recorded using a UV–Visible spectrophotometer (8543; Agilent Technologies Inc.) equipped with a temperature control unit (89090A; Agilent Technologies Inc.). To prepare the oxy Hb-HSA_*4*_, O_2_ gas was flowed to the PBS (pH 7.4) of carbonyl Hb-HSA_*4*_ ([Hb]: ca. 10 μM) in an optical quartz cuvette (10 mm path length) sealed with a rubber septum under light (500 W halogen lamp) in an ice-water bath. Subsequently, N_2_ gas was flushed to the O_2_ complex solution, yielding deoxy Hb-HSA_*4*_. The O_2_ affinity (*P*_50_: O_2_-partial pressure where Hb is half-saturated with O_2_) and Hill coefficient (*n*) were determined using an automatic recording system for blood O_2_-equilibrium curves (Hemox-Analyzer; TCS Scientific Corp.) using PBS (pH 7.4) at 37°C. The sample was oxygenated by an increasing O_2_-partial pressure and deoxygenated by flowing with N_2_. The visible absorption spectral changes, O_2_-euqilibrium curves, *P*_50_, and *n* of XLHb-HSA_*3*_ were also measured using the same procedures.

### Animal Experiments

All animal experiments were approved by the Animal Care and Use Committee of Sojo University and Kumamoto University. Care and handling of the animals were performed in accordance with the guidelines, principles, and procedures for the care and use of laboratory animals of Sojo University and Kumamoto University. All male Wistar rats were purchased from Kyudo Co. Ltd. and were maintained under conventional housing conditions. The animals were acclimated for 1 week before the experiments.

### Blood Circulation Experiments

The 125-Iodinated Hb-HSA_*4*_ and XLHb-HSA_*3*_ were prepared using our previously reported procedures [24,28]. The sample was diluted with non-labeled Hb-HSA_*4*_ and XLHb-HSA_*3*_ to adjust the protein concentration. Wistar rats were anesthetized with diethylether. The body weights were 253 ± 8 g for Hb-HSA_*4*_ group and 244 ± 7 g for XLHb-HSA_*3*_ group. Then the cluster solution was injected intravenously via a tail vein (Hb-HSA_*4*_, 50 mg/kg; XLHb-HSA_*3*_, 40 mg/kg) (0.2 mL/100 g, 1.5 × 10^6^ cpm/rat, *n* = 6). At the time-points of 3, 10, 30 min, and 1, 3, 6, 12, and 24 h after the infusion, 200 μL of blood was collected from the lateral tail vein using a heparinized syringe. After removal of the blood cell components by centrifugation (3000 rpm, 10 min), the ^125^I concentrations in the plasma were ascertained by measuring their radioactivity using an automatic gamma counter (2480 WIZARD2; PerkinElmer Inc.). Acid precipitability of the recovered radionuclide was confirmed using trichloroacetic acid. At the end of the experiments, the rats were anesthetized with diethylether and were euthanized by hemorrhage. Their vital organs (liver, kidney, spleen, lung, heart, and pancreas) were isolated, and their weights were measured. Urine and feces were also collected at fixed intervals in a metabolic cage. The radioactivity of the excised organs, urine, and feces were determined using a gamma counter as described above. As reference groups, identical experiments were conducted using the ^125^I-labeled samples in the same protocols [Hb group (270 ± 9 g, 10 mg/kg infusion, *n* = 6), HSA group (260 ± 11 g, 30 mg/kg infusion, *n* = 6), HSA group (268 ± 7 g, 40 mg/kg infusion, *n* = 4), and Hb-HSA_*3*_ group (269 ± 5 g, 40 mg/kg infusion, *n* = 6)]. The data of these groups were partially described in our previous report [24]. Furthermore, comparison experiments were conducted to measure the radioactivity of the vital organs at 1 h after the infusion [HSA group (262 ± 13 g, 30 mg/kg infusion, *n* = 6), HSA group (256 ± 11 g, 40 mg/kg infusion, *n* = 6), Hb-HSA_*3*_ group (274 ± 8 g, 40 mg/kg infusion, *n* = 6), Hb-HSA_*4*_ group (239 ± 8 g, 50 mg/kg infusion, *n* = 6), and XLHb-HSA_*3*_ group (243 ± 4 g, 40 mg/kg infusion, *n* = 6)].

### Data Analysis

Pharmacokinetic analyses of the samples were conducted using a non-compartment model. Pharmacokinetic parameters were calculated using the moment analysis program available with Microsoft Excel (Microsoft Corp.). Data are shown as the mean ± SD for the indicated number of animals. Significant differences between groups were inferred using two-tailed unpaired Student *t*-tests. Probability *p* < 0.05 was inferred as statistically significant.

## Results and Discussion

### Synthesis of Hb-HSA_*4*_ and XLHb-HSA_*3*_

**Fig 2:**
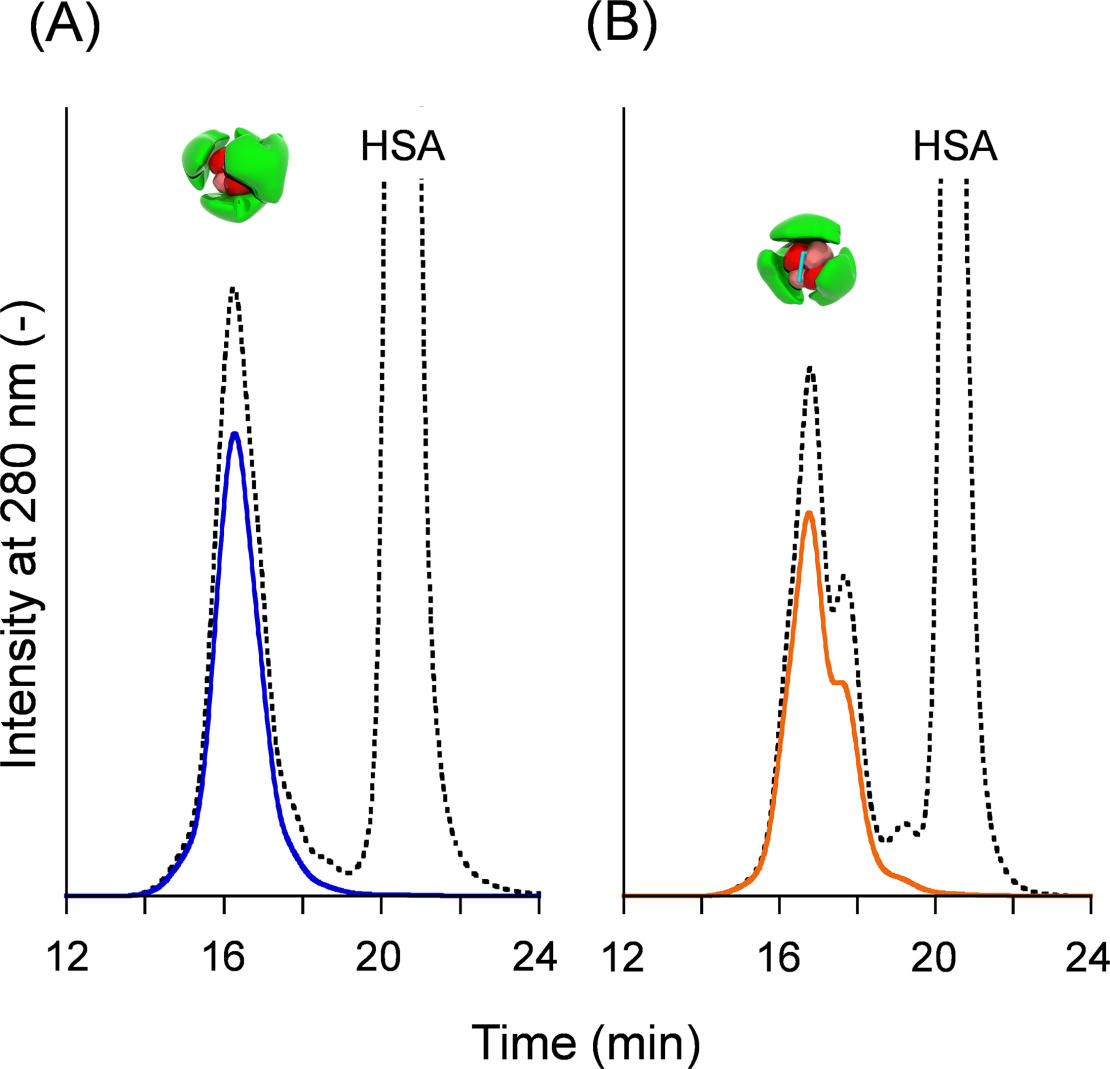
**SEC profiles of (A) Hb-HSA**_***4***_ **and (B) XLHb-HSA**_***3***_. Dotted black lines are elution curves of the reaction mixture. Solid lines are elution curves of purified clusters.

Fundamentally, Hb-HSA_*4*_ was prepared using the same procedure as that used for Hb-HSA_*3*_ with some modifications [21]. First, 40-fold molar excess SMCC was reacted with Hb in PBS (pH 7.4). The maleimide-activated-Hb (MA-Hb) was added dropwise into the HSA solution (HSA/MA-Hb = 20, mol/mol), followed by stirring for 14 h at 4°C. Both the SMCC/Hb and HSA/MA-Hb ratios are double the synthesis of Hb-HSA_*3*_. Size exclusion chromatography (SEC) of the reaction mixture demonstrated an approximately single peak at the high molecular weight region (14‒19 min elution time) (Fig 2A). Unreacted Hb (ca. 22 min) was not detectable. The position of the peak top (16.3 min) coincided well with that of Hb-HSA_*4*_ hetero-pentamer [21]. The major product was purified by gel filtration chromatography (GFC); the excess HSA was removed. Based on the protein assay and Hb assay, the average binding number of HSA on one Hb was found to be 4.0 ± 0.2.

**Fig 3:**
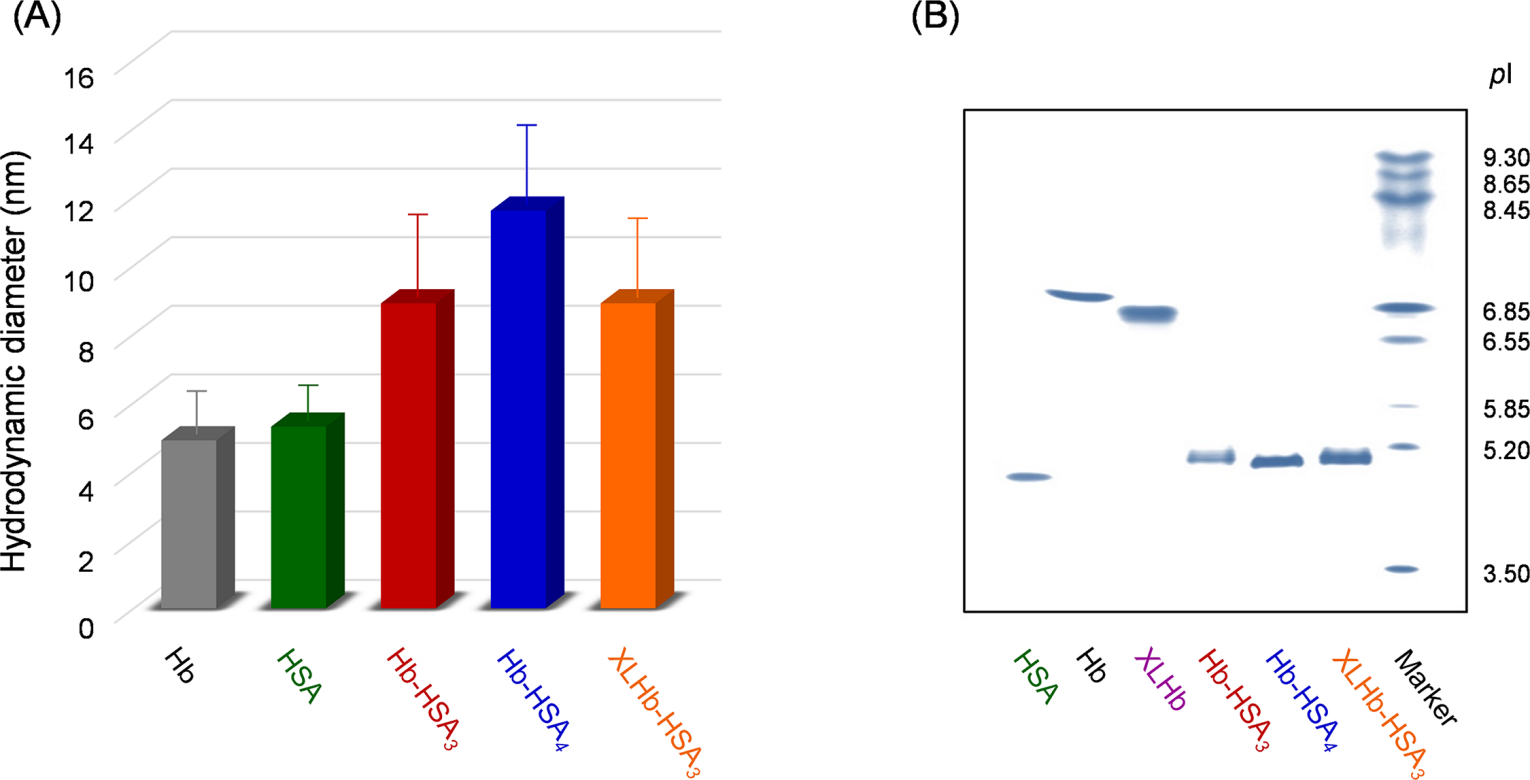
Diameters and IEF patterns of hemoglobin-albumin clusters. (A) Hydrodynamic diameters of Hb-HSA_*3*_, Hb-HSA_*4*_, and XLHb-HSA_*3*_ measured using DLS. (B) IEF patterns of Hb-HSA_*3*_, Hb-HSA_*4*_, and XLHb-HSA_*3*_.

The bovine Hb processes two sulfhydryl groups of Cys-93(β) residues in the central cavity (Fig 1B) [25]. Therefore, the site-specific reaction of α,ω-bismaleimide reagent with Hb would enable us to make an intramolecular bridge in the protein. Because the distance of two Cys-93(β) residues is estimated as 21.6 Å based on crystal structural data [25], we choose 1,11-bismaleimido-triethyleneglycol (BMTEG) (18.3 Å molecular length). The SDS-PAGE of the resultant mixture exhibited two bands with the same intensity at 16 kDa and 32 kDa, which respectively correspond to the *α*-subunit monomer and crosslinked ββ-subunit dimer (S1 Fig). The number of cysteinyl thiols per Hb decreased from 2.0 to 0.1 [27]. These results imply that two Cys-93(β) residues are connected intramolecularly by BMTEG, yielding ββ-crosslinked Hb (Fig 1B). Isoelectric focusing (IEF) electrophoresis analysis showed that the isoelectric point (*p*I) of XLHb was slightly lower than the value of naked Hb (*p*I: 7.0) (Fig 3B). Acharya *et al*. reported that crosslinked Hbs [ββ-Hb with bis(maleimidophenyl)-PEG2000 and αα-Hb with bis(3,5-dibromosalicyl)fumarate] demonstrated the same electrophoretic mobility with unmodified Hb [7,10]. Presumably, site-specific crosslinking of Cys-93(β)s by BMTEG influences the orientations of ε-amino groups of Lys residues on the molecular surface, thereby reducing the *p*I value.

The XLHb-HSA_*3*_ was prepared using the same procedure for Hb-HSA_*3*_ using XLHb [21]. The SEC profile of XLHb-HSA_*3*_ resembled that of Hb-HSA_*3*_ (Fig 2B) [21]. The average binding number of HSA on one XLHb was ascertained as 2.9 ± 0.2.

Molecular dimensions of these protein clusters were estimated from dynamic light scattering (DLS) measurements. The hydrodynamic diameters increased with the number of HSA units: 8.9 nm for Hb-HSA_*3*_, 8.9 nm for XLHb-HSA_*3*_, and 11.6 nm for Hb-HSA_*4*_ (Fig 3A). The Hb-HSA_*4*_ (molecular weight: ca. 332 kDa) is larger than the others having three HSA entities. The IEF pattern revealed that the *p*I values of the clusters (*p*I: 5.0−5.1) were close to that of HSA (*p*I: 4.9) (Fig 3B). It is noteworthy that the covalent wrapping of Hb with three-fold HSAs is sufficient to mask the surface charge of the Hb core (*p*I: 7.0).

### O_2_-Binding Properties

Visible absorption spectra of Hb-HSA_*4*_ and XLHb-HSA_*3*_ in PBS solution under N_2_, O_2_, and CO atmospheres (deoxy, oxy, and carbonyl forms, respectively) were identical to the corresponding spectra of native Hb and Hb-HSA_*3*_ (S2 Fig and S1 Table) [29]. The O_2_ affinities (*P*_50_: O_2_-partial pressure where Hb is half-saturated with O_2_) and cooperativity coefficients (Hill coefficient; *n*) were determined from the O_2_-binding equilibrium curves. The *P*_50_ of Hb-HSA_*4*_ (9 Torr) was significantly lower than the value of Hb (*P*_50_ = 23 Torr) (Table 1). The Hill coefficient also decreased from 2.6 to 1.5. The reductions of *P*_50_ and *n* were equivalent to those observed for Hb-HSA_*3*_. This report describes two feasible reasons for the high O_2_-affinity and low cooperativity of Hb-HSA_*3*_, which were explained in earlier reports [20,23]. First is the blocking of a sulfhydryl group of Cys-93(β) in Hb by the maleimide terminal of free SMCC. Chemical modification of Cys-93(β), which is located beside the proximal base (His-92) of the heme, is known to enhance the O_2_ affinity [9,11,30] and is known to restrict the quaternary structure of Hb from the Tense (T)-state to a Relaxed (R)-state [30]. The second reason is the modifications of ε-amino groups of Lys residues on Hb by the succinimide terminal of SMCC. They are necessary to create the cluster, whereas the chemical modification of Lys affects the O_2_ affinity and decreases the Hill coefficient of Hb [8,31,32]. Identical O_2_-binding parameters of Hb-HSA_*4*_ and Hb-HSA_*3*_ imply that the central Hb conformations are almost identical, independent of the binding number of HSA.

However, XLHb-HSA_*3*_ showed moderately low *P*_50_ and *n* (6 Torr, 1.0) with respect to Hb-HSA_*3*_ and Hb-HSA_*4*_. The Hill coefficient of 1.0 indicates a lack of cooperatively. The two sulfhydryl groups of Cys-93(β)s in Hb were bridged securely, thereby strongly constraining the available motion of quaternary structure of the XLHb core. The high O_2_-affinity variant may be advantageous as a transfusion-related medication. Another possible reason of the side-effect by Hb derivatives is overoxygenation [33–36]. The small Hb injected into the bloodstream facilitates the diffusive O_2_ transport to the blood vessel wall, initiating overoxygenation of the surrounding tissues. An autoregulatory O_2_-sensing system responds by constricting the blood vessel and reduces the available surface area for O_2_ transport. These physiological responses engender vasoconstriction. The high O_2_-affinity (low *P*_50_) avoids early O_2_ offloading on the arterial vessels and may be beneficial for O_2_ release in the capillaries [34–36].

**Table 1:** O_2_-binding parameters of Hb-HSA_*3*,_ Hb-HSA_*4*,_ and XLHb-HSA_*3*_ in PBS solution (pH 7.4) at 37°C.

Hemoproteins	*P*_50_ (Torr)	*n*	*k*_ox_ (h^-1^)
Hb ^a^	23	2.6	0.037
Hb-HSA_3_ ^a^	9	1.5	0.035
Hb-HSA_4_	9	1.5	0.039
XLHb-HSA_*3*_	6	1.0	0.033

^a^ Ref. 21.

### Blood Circulation and Tissue Distribution

**Fig 4:**
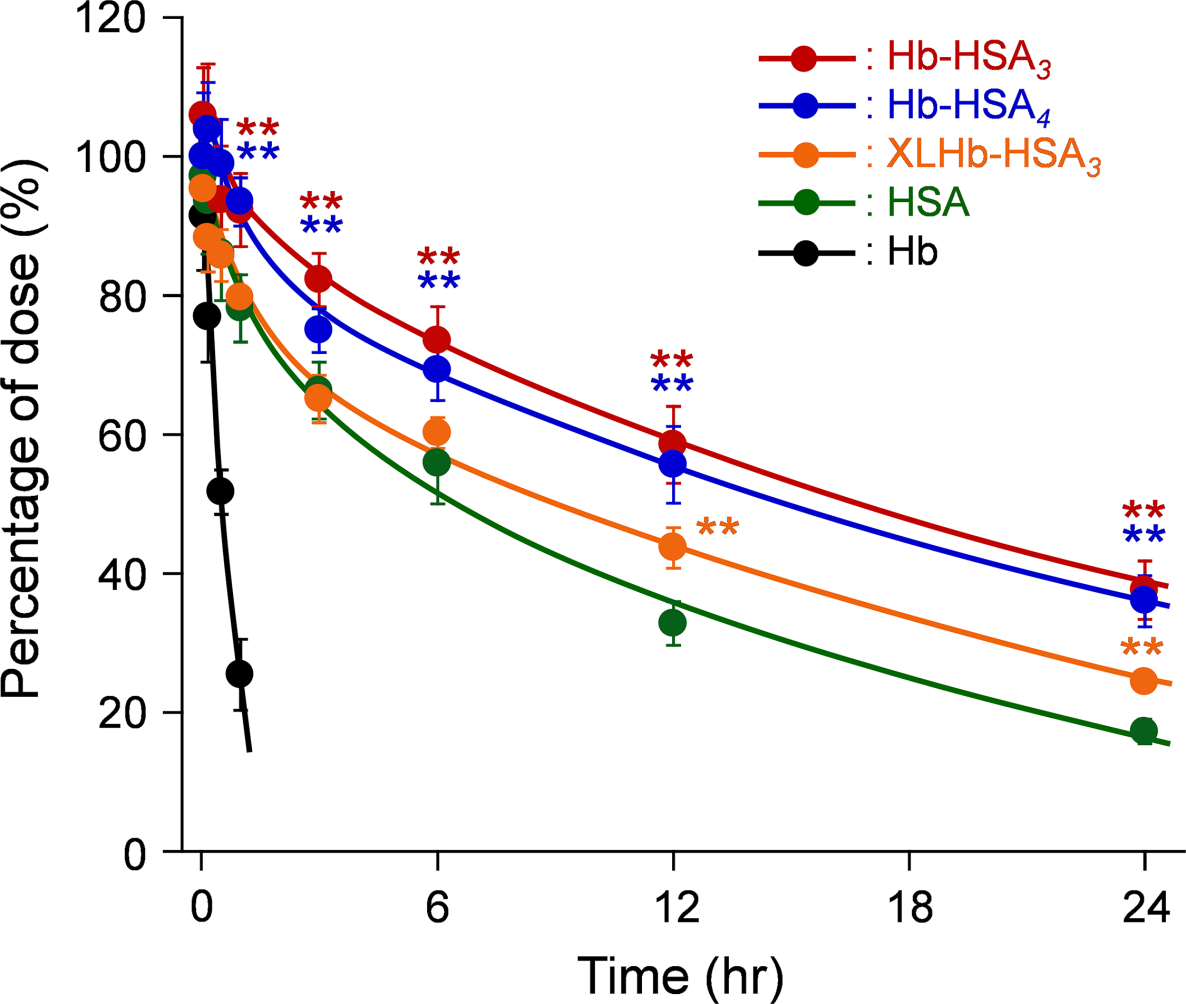
Blood retention of hemoglobin-albumin clusters. Relative plasma concentrations of ^125^I-labeled Hb-HSA_*3*_, Hb-HSA_*4*_, XLHb-HSA_*3*_, and HSA after intravenous administration to rats. Each data point represents the mean ± SD (*n* = 6). **p < 0.01 vs. HSA.

The ^125^I-labeled cluster solutions were injected into rats to observe the blood retention and tissue distribution. The ^125^I-labeled Hb was eliminated immediately from the circulation (Fig 4) [half-life (*T*_50_): 0.53 h] [24]. In contrast, time courses of plasma concentration of the clusters demonstrated slower kinetics. Blood circulation parameters were ascertained using a non-compartment model (Table 2). The *T*_50_ of Hb-HSA_*3*_ (18.5 ± 1.7 h) was 1.7-fold longer than that of HSA (*T*_50_ = 11.0 ± 0.67 h) [24]. This greater length is attributable to the decreases of clearance (CL) and volume of distribution in a steady state (*V*_dss_). The negative surface net charge and large molecular size of Hb-HSA_*3*_ suppress the movement to the extravascular space and renal filtration. In view of these findings, we anticipated that Hb-HSA_*4*_ bearing four HSA units, a large-size variant, might show longer blood retention. Nonetheless, the result fell short of our expectations. All parameters of Hb-HSA_*4*_ were comparable to those of Hb-HSA_*3*_ (Table 2). The kinetic profiles of HSA with a 30 mg/kg infusion and 40 mg/kg infusion were almost identical (S3 Fig and Table 2). This result shows that the quantity of the protein administrated does not influence the blood circulation parameters in our experimental models. We concluded that the increase of HSA binding number on Hb (increase of molecular weight of the cluster) is ineffective to extend the circulation persistence. In other words, the three-fold HSA conjugation to Hb is sufficient to retain the cluster molecules in the bloodstream for a long period.

**Table 2:** Blood circulation parameters of ^125^I-labeled Hb-HSA_*3*,_ Hb-HSA_*4*,_ XLHb-HSA_*3*_, and HSA after intravenous administration to rats (*n* = 6).

	*T*_50_ (h)	MRT (h)	CL_tot_ (mL/h)	*V*_dss_ (mL)	AUC (% of dose/mL h)
Hb ^a^	0.53 ± 0.1	0.74 ± 0.14	16.5 ± 2.4	12.0 ± 1.3	6.2 ± 0.9
HSA ^a^^,^^b^	11.0 ± 0.67	15.0 ± 0.88	0.95 ± 0.07	14.3 ± 1.4	105.5 ± 8.2
HSA ^c^	10.8 ± 0.8	14.6 ± 1.1	0.94 ± 0.11	13.6 ± 1.1	107.9 ± 12.5
Hb-HSA_*3*_ ^a^	18.5 ± 1.7**	26.3 ± 2.5**	0.50 ± 0.07**	13.0 ± 0.74	203.4 ± 23.2**
Hb-HSA_*4*_	19.1 ± 1.6**	27.1 ± 2.4**	0.48 ± 0.06**	13.0 ± 0.9	210.0 ± 26.9**
XLHb-HSA_*3*_	13.8 ± 0.4**	19.6 ± 0.5**	0.68 ± 0.03**	13.4 ± 0.6	146.4 ± 7.2**

*T*_50_, half-life; MRT, mean residence time; CL_total_, clearance; *V*_dss_, volume of distribution in a steady state; AUC, area under the concentration-time curve.

^a^ Ref. 24.

^b^ 30 mg/kg dose.

^c^ 40 mg/kg dose. Each value represents the mean ± SD (*n* = 6).

**p < 0.01 vs. HSA^*b*^.

In contrast, the *T*_50_ of XLHb-HSA_*3*_ (13.8 ± 0.4 h) was rather shorter than that of Hb-HSA_3_, despite their molecular weights, diameters, and surface charges are nearly equal. It appears to be attributable to different morphology of the cluster. The intramolecular crosslinking of Cys-93(β)s of Hb might change the reactivity of ε-amino groups of Lys residues around the BMTEG bridge. Results show that the binding positions of HSA units on XLHb become partially different from those of Hb-HSA_*3*_, yielding a different-shape variant. In fact, our image processing and 3D reconstruction based on transmission electron microscopy data of Hb-HSA_*3*_ revealed that Lys-82(β_1_), which is located between the two Cys-93(β)s of Hb, is a potential binding partner of Cys-34 of HSA [20]. We presumed that the change of molecular shape of the cluster might cause measurable alteration of the blood circulation kinetics.

**Fig 5:**
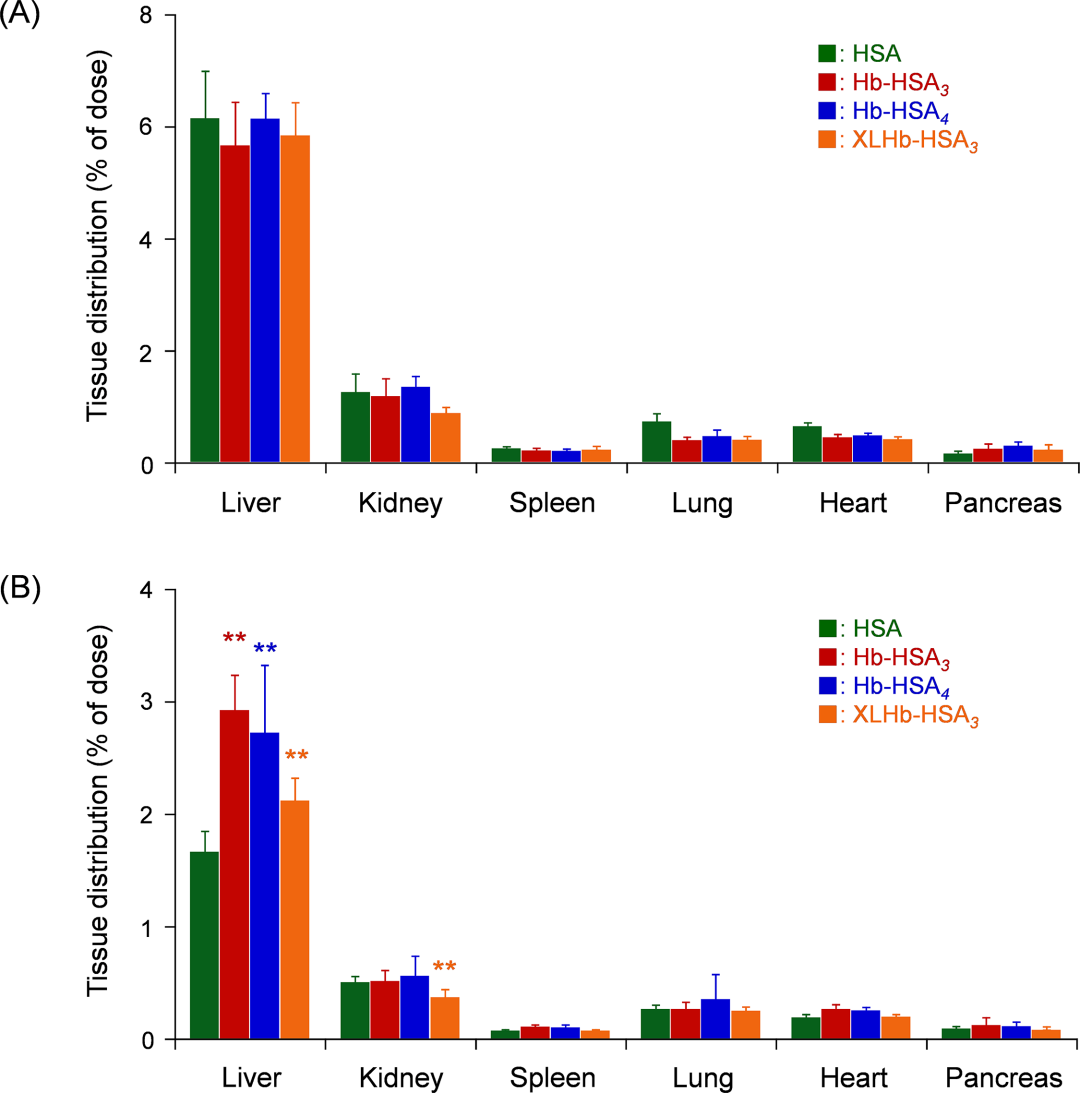
Tissue distribution of hemoglobin-albumin clusters. Tissue (vital organs) distribution of ^125^I radioactivity (% of dose) (A) at 1 h and (B) at 24 h after intravenous administration of ^125^I-labeled Hb-HSA_*3*_, Hb-HSA_*4*_, XLHb-HSA_*3*_, and HSA to rats. Each bar shows the mean ± SD (*n* = 6). **p < 0.01 vs. HSA.

The tissue distribution of the clusters in vital organs (liver, kidney, spleen, lung, heart, and pancreas) was determined at 1 h and 24 h after the administration (Fig 5). No correlation was found among the three cluster groups and the HSA group at 1 h after infusion (Fig 5A). However, marked differences of the accumulated quantities were observed in the liver after 24 h (Fig 5B). The high hepatic distributions of Hb-HSA_*3*_, Hb-HSA_*4*_, and XLHb-HSA_*3*_ compared to HSA are ascribed to their large molecular sizes. These high distributions are consistent with data reported by Rennen *et al*. showing predominantly uptake of large proteins in the liver [37]. Among the three clusters, small distribution in liver was found in XLHb-HSA_*3*_ group. We inferred that such a difference is also caused by the variation of the cluster molecular structure.

**Fig 6:**
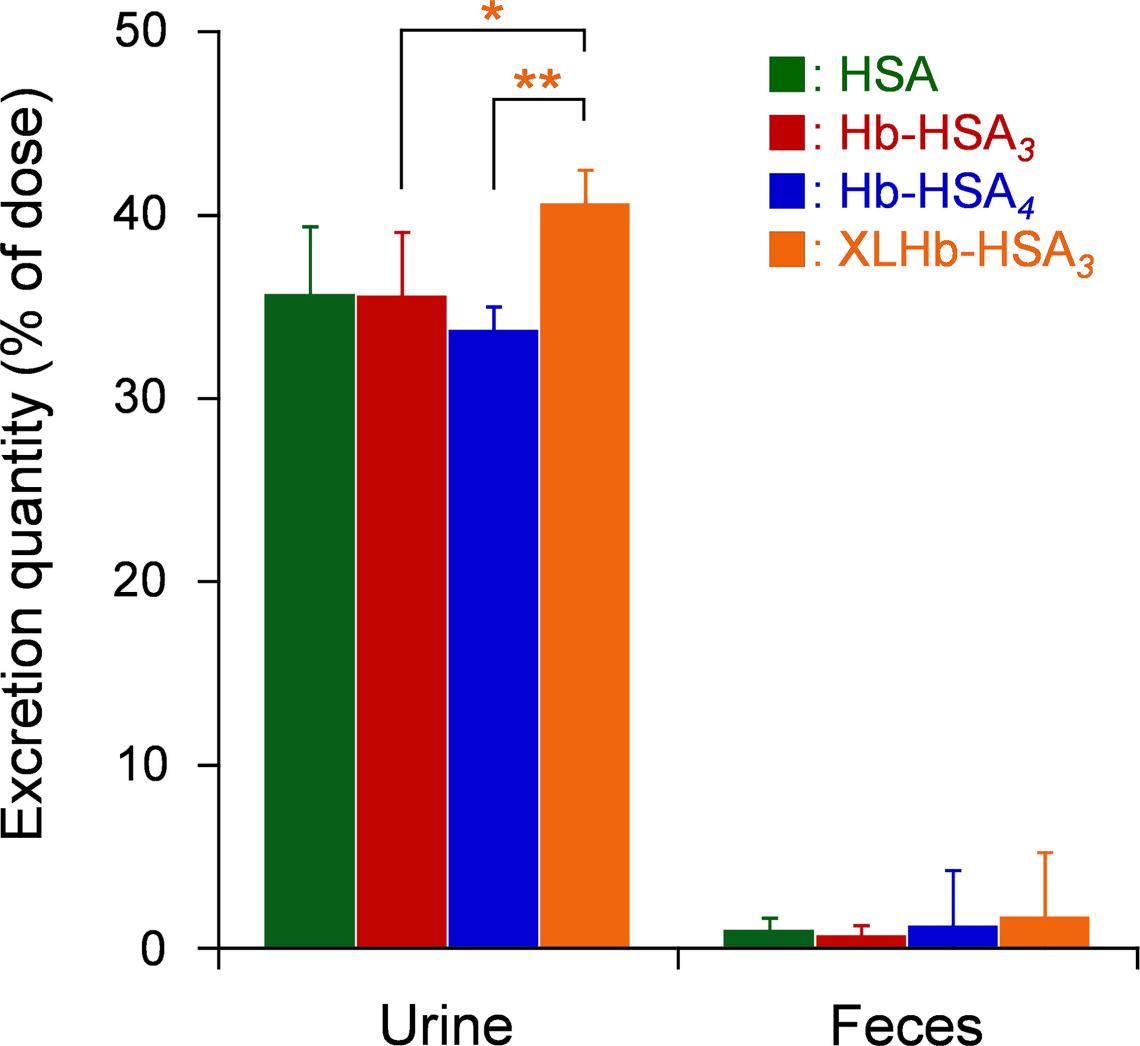
Urinary and fecal excretions. Urinary and fecal excretions of ^125^I radioactivity (% of dose) within 24 h after intravenous administration of ^125^I-labeled Hb-HSA_*3*_, Hb-HSA_*4*_, XLHb-HSA_*3*_, and HSA to rats. Each bar shows the mean ± SD (*n* = 6). *p < 0.05 vs. Hb-HSA_*3*_, **p < 0.01 vs. Hb-HSA_*4*_.

Total ^125^I activities of urine within 24 h were much higher than those of feces in all groups (Fig 6). The clusters are metabolized in the body and are excreted into urine, which is the same excretion pathway of HSA. The excreted ^125^I amount in urine of XLHb-HSA_*3*_ group was somewhat higher than those of other groups. Results show that XLHb-HSA_*3*_ is metabolized faster than Hb-HSA_*3*_ and Hb-HSA_*4*_, and suggest that the molecular structure influences the catabolism of protein clusters in the body.

## Conclusion

Covalent wrapping of Hb or XLHb with three or four HSAs generates various core–shell protein cluster conjugates. The Hb-HSA_*4*_ diameter was larger than those of the others enfolded by three HSA units. Nevertheless, increasing of the HSA binding number has no marked influence on the O_2_-binding property and blood circulation. The XLHb-HSA_*3*_ showed high O_2_-affinity (*P*_50_ = 6 Torr) because the Cys-93(β) residues of Hb are crosslinked intramolecularly. The superior blood circulation properties of all clusters compared to native Hb are attributed to their negative surface net charges and larger molecular sizes. It is noteworthy that the circulation parameters of XLHb-HSA_*3*_ are close to those of HSA. This variant was metabolized faster than Hb-HSA_*3*_ and Hb-HSA_*4*_. Site-specific Cys-93(β) crosslinking of the Hb core may alter the chemical reactivity of Lys residues around the bridging site, thereby forming a different spatial conformation of the cluster and causing a useful reduction of the blood circulation. Our results imply that the molecular structure is a factor that might affect the *in vivo* circulation behavior of the cluster. The Hb-HSA_*4*_ and XLHb-HSA_*3*_ will become unique RBC substitutes that may serve as a relay product enabling resuscitation of blood loss patients.

## Supporting Information

S1 Fig SDS-PAGE of XLHb. (TIF)

S2 Fig UV-vis. absorption spectral change of XLHb-HSA_*3*_. In PBS solution at 25°C.
(TIF)

S3 Fig Blood retention of HSA. Relative plasma concentrations of ^125^I-labeled HSA after intravenous 30 mg/kg infusion (*n* = 6) and 40 mg/kg infusions (*n* = 4) to rats. Each data point represents the mean ± SD.
(TIF)

S1 Table Visible absorption spectral data of Hb-HSA_*4*_ and XLHb-HSA_*3*_. In PBS solution (pH 7.4) at 25°C
(TIF)
