# Supplementary figures and images for: Influence of Molecular Structure on O2-Binding Properties and Blood Circulation of Hemoglobin‒Albumin Clusters
Source: PLoS One. 2016 Feb 19;11(2):e0149526. doi: 10.1371/journal.pone.0149526 (PMC4760709; doi:10.1371/journal.pone.0149526)

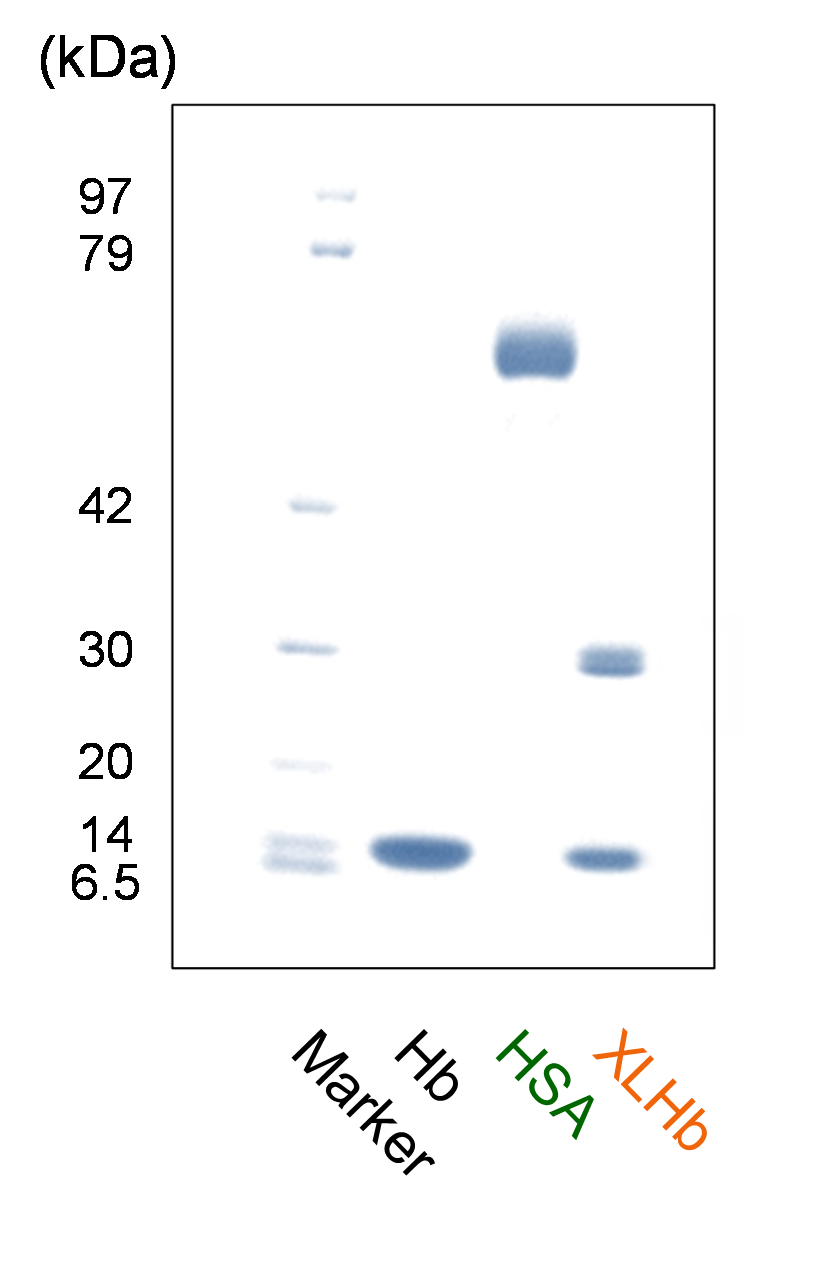

Supplement: S1 Fig — (TIF) [file pone.0149526.s001.tif]

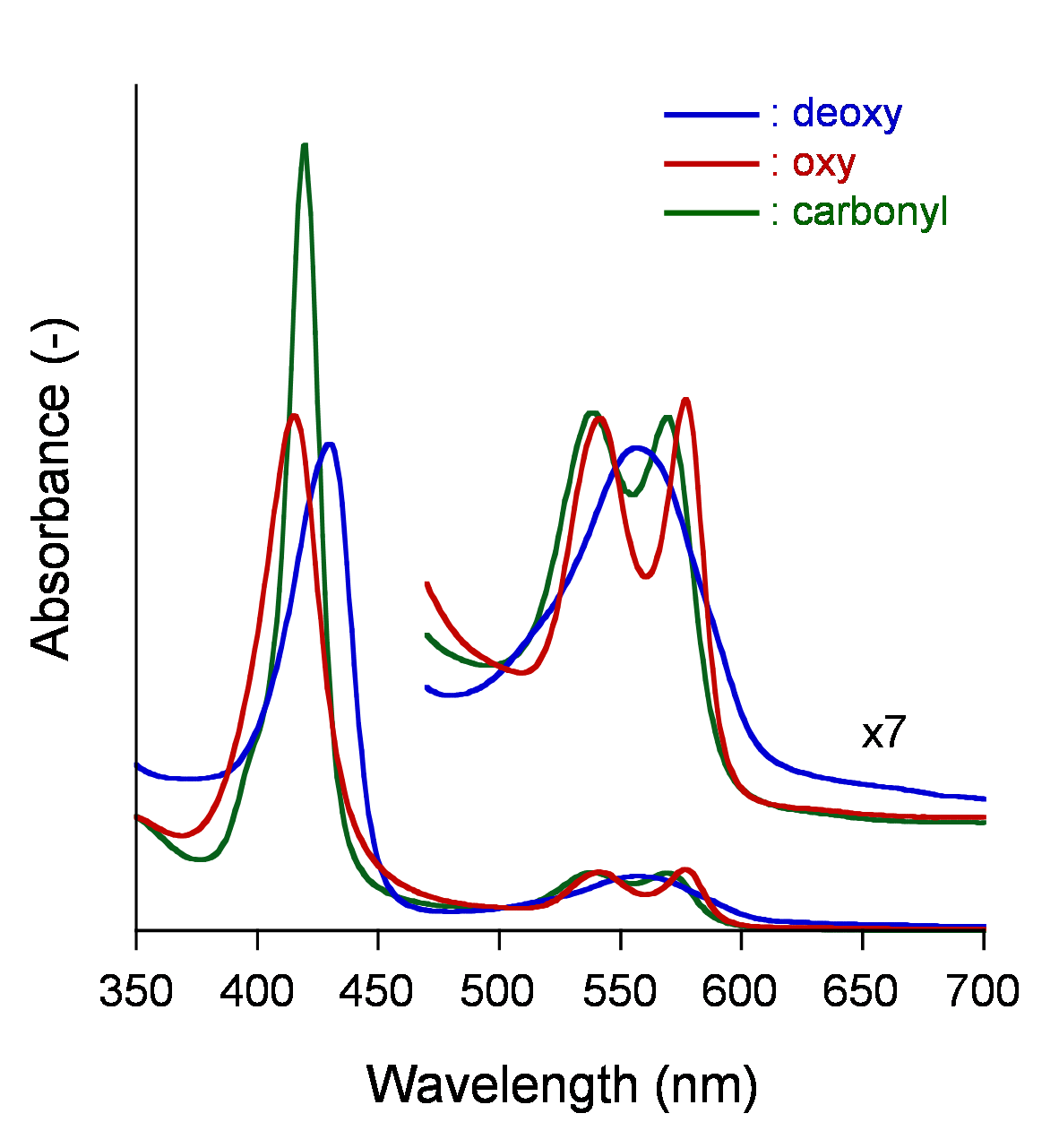

Supplement: S2 Fig — In PBS solution at 25°C. (TIF) [file pone.0149526.s002.tif]

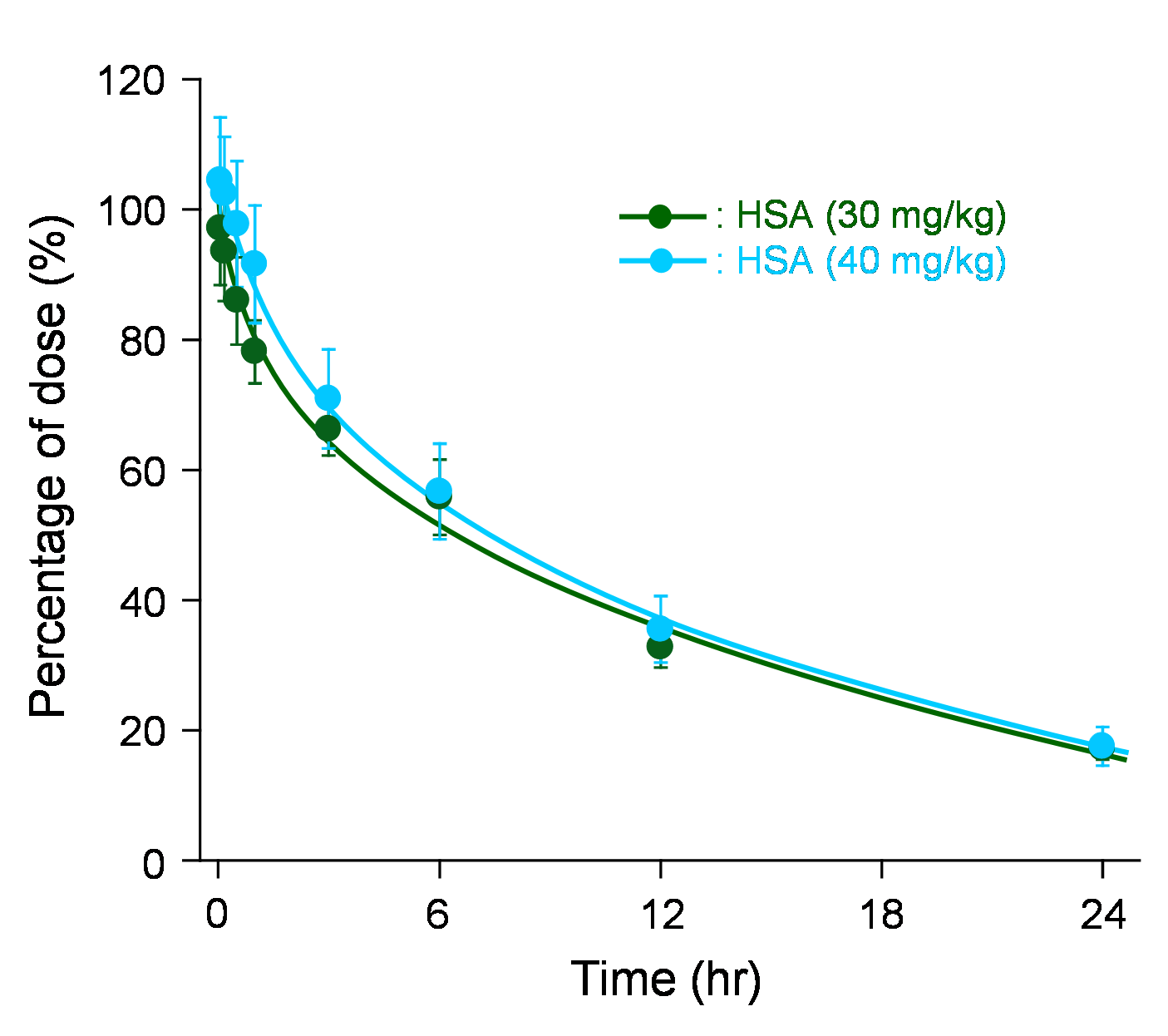

Supplement: S3 Fig — Relative plasma concentrations of 125I-labeled HSA after intravenous 30 mg/kg infusion (n = 6) and 40 mg/kg infusions (n = 4) to rats. Each data point represents the mean ± SD. (TIF) [file pone.0149526.s003.tif]

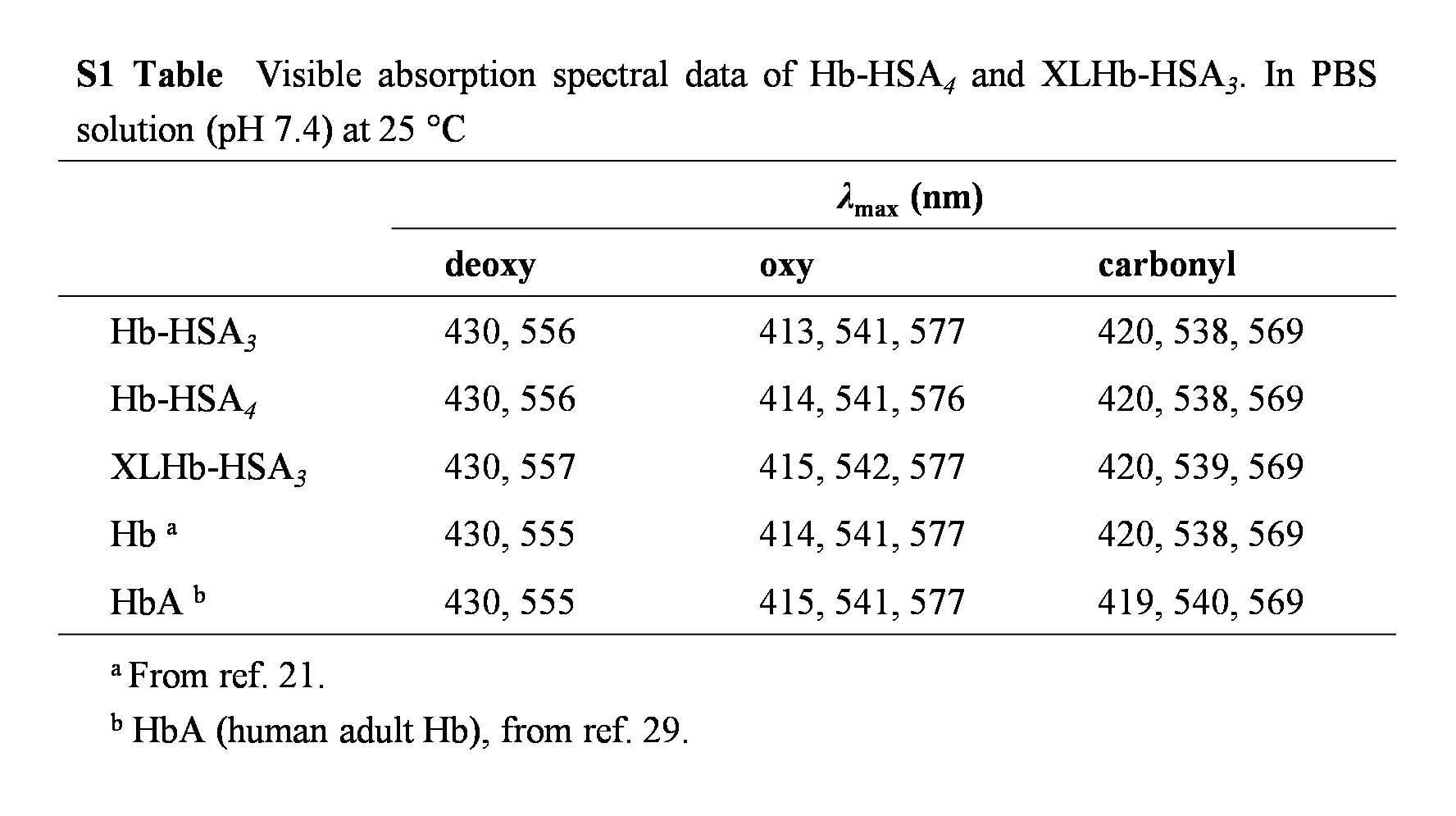

Supplement: S1 Table — In PBS solution (pH 7.4) at 25°C (TIF) [file pone.0149526.s004.tif]
